# Supplementary material for: HIVprotI: an integrated web based platform for prediction and design of HIV proteins inhibitors
Source: J Cheminform. 2018 Mar 9;10:12. doi: 10.1186/s13321-018-0266-y (PMC5845081; doi:10.1186/s13321-018-0266-y)
Supplement: Supplementary file 2 — Additional file 2. Source code of HIVProtI web server. [file 13321_2018_266_MOESM2_ESM.zip › HIVprotI_Source-code/hivprotI/weka-3-6-6/doc/weka/datagenerators/RegressionGenerator.html]

RegressionGenerator


---


|  |  |  |
| --- | --- | --- |
| **Overview** \| **Package** \| **Class** \| **Tree** \| **Deprecated** \| **Index** \| **Help** \| **Weka's home** | |  |
| **PREV CLASS**   **NEXT CLASS** | **FRAMES**    **NO FRAMES**     **All Classes** |  |
| SUMMARY: NESTED \| FIELD \| CONSTR \| METHOD | DETAIL: FIELD \| CONSTR \| METHOD |  |


---


## weka.datagenerators Class RegressionGenerator

```
java.lang.Object
  weka.datagenerators.DataGenerator
      weka.datagenerators.RegressionGenerator
```

**All Implemented Interfaces:**: java.io.Serializable, OptionHandler, Randomizable, RevisionHandler

**Direct Known Subclasses:**: MexicanHat

---

``` public abstract class RegressionGenerator extends DataGenerator ```

Abstract class for data generators for regression classifiers.

Example usage as the main of a datagenerator called RandomGenerator:

```
 public static void main(String[] args) {
   try {
     DataGenerator.makeData(new RandomGenerator(), args);
   } 
   catch (Exception e) {
     e.printStackTrace();
     System.err.println(e.getMessage());
   }
 }
```

**Version:**
:   $Revision: 1.3 $

**Author:**
:   FracPete (fracpete at waikato dot ac dot nz)

**See Also:**: Serialized Form

---

**Constructor Summary**

`RegressionGenerator()`
  
          initializes the generator with default values


| **Method Summary** | |
| --- | --- |
| `int` | `getNumExamples()`             Gets the number of examples, given by option. |
| `java.lang.String[]` | `getOptions()`             Gets the current settings of the classifier. |
| `java.util.Enumeration` | `listOptions()`             Returns an enumeration describing the available options. |
| `java.lang.String` | `numExamplesTipText()`             Returns the tip text for this property |
| `void` | `setNumExamples(int numExamples)`             Sets the number of examples, given by option. |
| `void` | `setOptions(java.lang.String[] options)`             Sets the options. |

**Methods inherited from class weka.datagenerators.DataGenerator**

`debugTipText, defaultOutput, defineDataFormat, formatTipText, generateExample, generateExamples, generateFinished, generateStart, getDatasetFormat, getDebug, getNumExamplesAct, getOutput, getRandom, getRelationName, getSeed, getSingleModeFlag, makeData, outputTipText, randomTipText, relationNameTipText, seedTipText, setDatasetFormat, setDebug, setOutput, setRandom, setRelationName, setSeed`

**Methods inherited from class java.lang.Object**

`equals, getClass, hashCode, notify, notifyAll, toString, wait, wait, wait`

**Methods inherited from interface weka.core.RevisionHandler**

`getRevision`

**Constructor Detail**

### RegressionGenerator

```
public RegressionGenerator()
```

:   initializes the generator with default values


**Method Detail**

### listOptions

```
public java.util.Enumeration listOptions()
```

:   Returns an enumeration describing the available options.

    :   **Specified by:**: `listOptions` in interface `OptionHandler` **Overrides:**: `listOptions` in class `DataGenerator`
    :   **Returns:**: an enumeration of all the available options.

---


### setOptions

```
public void setOptions(java.lang.String[] options)
                throws java.lang.Exception
```

:   Sets the options.

    :   **Specified by:**: `setOptions` in interface `OptionHandler` **Overrides:**: `setOptions` in class `DataGenerator`
    :   **Parameters:**: `options` - the options **Throws:**: `java.lang.Exception` - if invalid option

---


### getOptions

```
public java.lang.String[] getOptions()
```

:   Gets the current settings of the classifier.

    :   **Specified by:**: `getOptions` in interface `OptionHandler` **Overrides:**: `getOptions` in class `DataGenerator`
    :   **Returns:**: an array of strings suitable for passing to setOptions **See Also:**: `DataGenerator.removeBlacklist(String[])`

---


### setNumExamples

```
public void setNumExamples(int numExamples)
```

:   Sets the number of examples, given by option.

    :   **Parameters:**: `numExamples` - the new number of examples

---


### getNumExamples

```
public int getNumExamples()
```

:   Gets the number of examples, given by option.

    :   **Returns:**: the number of examples, given by option

---


### numExamplesTipText

```
public java.lang.String numExamplesTipText()
```

:   Returns the tip text for this property

    :   **Returns:**: tip text for this property suitable for displaying in the explorer/experimenter gui


---


|  |  |  |
| --- | --- | --- |
| **Overview** \| **Package** \| **Class** \| **Tree** \| **Deprecated** \| **Index** \| **Help** \| **Weka's home** | |  |
| **PREV CLASS**   **NEXT CLASS** | **FRAMES**    **NO FRAMES**     **All Classes** |  |
| SUMMARY: NESTED \| FIELD \| CONSTR \| METHOD | DETAIL: FIELD \| CONSTR \| METHOD |  |


---
